# Initial puffing behaviors and subjective responses differ between an electronic nicotine delivery system and traditional cigarettes

**DOI:** 10.1186/1617-9625-12-17

**Published:** 2014-10-10

**Authors:** Kaila J Norton, Kristie M June, Richard J O’Connor

**Affiliations:** 1 Department of Health Behavior, Roswell Park Cancer Institute, Elm and Carlton Streets, 14263 Buffalo, NY, USA

**Keywords:** Smoking, Behavior, Subjective effects

## Abstract

**Background:**

Electronic nicotine delivery systems (ENDS) present an emerging issue for tobacco control and data on product use behaviors are limited.

**Methods:**

Participants (N = 38 enrolled; N = 16 compliant) completed three lab visits over 5 days and were asked to abstain from regular cigarettes for 72 hours in favor of ENDS (Smoke 51 TRIO – 3 piece, First Generation with 11 mg/ml filters). Lab visits included measurement of exhaled carbon monoxide (CO) and salivary cotinine concentration, questionnaire measures of regular cigarette craving after the 72 hour abstinence, and subjective product effects. Participants used a topography device to record puff volume, duration, flow rate, and inter-puff interval.

**Results:**

Analyses revealed significant differences across products in puff count, average volume, total volume and inter-puff interval, with ENDS broadly showing a more intensive smoking pattern. Cigarette craving scores dropped significantly after smoking regular cigarettes, but not ENDS (p = .001), and subjective measures showed ENDS rated less favorably. CO boost, after ENDS use, decreased significantly (p < .001), and saliva cotinine significantly dropped between visits 1 and 3 (p < 0.001) after ENDS use relative to after cigarette smoking. For compliant and non-compliant participants, there was an average 82.0% [V1 - 16.1 cpd; V3 - 2.9 cpd] and average 73.9% [V1 - 20.3 cpd; V3 - 5.3 cpd] reduction in regular cigarette use per day during the ENDS trial period, respectively.

**Conclusions:**

The ENDS were smoked more intensively than own brand cigarettes, but delivered significantly less nicotineand were less satisfying. These findings have implications for the viability of certain ENDS as alternatives to cigarettes.

## Background

Electronic nicotine delivery systems (ENDS, commonly called ‘e-cigarettes’), nicotine-containing devices containing no tobacco, represent an emergent issue in tobacco control. A typical electronic cigarette consists of a cartridge containing nicotine dissolved in propylene glycol and/or glycerine, an atomizer, and a mouthpiece. When the user draws on the mouthpiece, a sensor detects the change in pressure and causes the atomizer to heat up and vaporize a solution of nicotine and propylene glycol and/or glycerine, which the user then inhales [1]. ENDS cartridges are sold with varying nicotine levels, and a number of websites sell e-liquids that allow users to refill their own cartridges or tanks [2,3].

Currently, ENDS are known to produce trace amounts of various toxic compounds such as tobacco specific nitrosamines, diethylene glycol, and aldehydes (formaldehyde, acetaldehyde and acrolein); though levels are far less than those in cigarettes [4-6]. Although design aspects of ENDS vary, leaky fluid contained in cartridge reservoirs, poor cartridge labeling, safety features that do not always function properly, and insufficient warnings on packaging have been noted as reasons for concern regarding consumer usage [2]. Tested ENDSs require significant vacuum pressure to aerosolize the solution in the cartridge, which is hypothesized to lead users to inhale harder and deeper to obtain the vapor from their ENDS [3]. While a tobacco cigarette produces smoke with a consistent density, ENDS vapor has been shown to decrease in density after the first 10 puffs on certain models, which also could lead users to modify their smoking behaviors to obtain more vapor [3].

Puffing topography data are needed to confirm that the vacuum pressure observation by Trtchounian and colleagues has relevance to consumer use. In a recent study by Hua et al. [7], puff and exhalation duration were compared for individuals using first generation ENDS and conventional cigarettes in YouTube videos. They found that puff duration was significantly longer for ENDS users (mean = 4.3 s) (N = 64) than for conventional cigarette users, and puff duration varied significantly among ENDS brands. Additionally, Goniewicz et al. [8], recruited 10 volunteers (aged 35 ± 20 years, 8 males) who used various brands and models of first generation ENDS for at least one month and measured their puffing topography. The average puffing topography were as follows (*M* ± *SD*): puff duration of 1.8 ± 0.9 s, intervals between puffs of 10 ± 13 s, puff volume 70 ± 68 ml, and number of puffs taken in one puffing session was 15 ± 6.

However, there is potential for ENDS to serve as a long-term alternative to cigarettes and even as a smoking cessation aid. Adkison et al. [9] examined data from the International Tobacco Control Four Country Survey, collected in 2010–11 among 5,939 current and former smokers in Canada, the US, the UK, and Australia, and found that among the 2.9% of the sample who currently used ENDS, 75.4% stated that they used ENDS to help cut down, and 85.1% used ENDS to help quit smoking. A study in Italy revealed ENDS use substantially decreased cigarette consumption without causing significant side effects among a group of smokers not intending to quit [10]. Additionally, Goniewicz et al. [11] reported that 41% of ENDS users utilized ENDS to quit smoking or reduce the harm associated with smoking. More recently, Caponnetto et al. [12] conducted a 9 visit, 12-month, randomized, controlled trial that evaluated smoking reduction/abstinence in 300 smokers (3 groups) not intending to quit; experimenting two different nicotine strengths of a popular e-cigarette model compared to its non-nicotine choice. In all three groups, smokers not intending to quit used of e-cigarettes to decrease cigarette consumption without significant side effects. In a separate study, the 16 mg Ruyan ENDS alleviated desire to smoke after overnight abstinence [13].

The current pilot study was initiated to examine initial reactions to use and puffing behaviors with first generation ENDS among inexperienced users.

## Methods

### Participants

Participants (N = 38) were recruited from February 2011-May 2012 via advertising in local newspapers (advertising did not mention the study focused on ENDS). Eligible participants were a minimum of 18 years of age, smoked at least 10 cigarettes daily, were not concurrently using other tobacco or nicotine products, had no use of ENDS in the last 30 days, reported no intention of quitting smoking within the next 30 days, had no medical contraindications for nicotine replacement products, were in good general health, and had no known sensitivity to propylene glycol and/or glycerin. Females who reported they might be pregnant or planned to become pregnant during the study were excluded from participation. We selected those who had not used ENDS in the past 30 days because we were primarily interested in initial reactions to the product among triers, rather than experienced ENDS users.

### Study design & procedures

The study design is illustrated in Table 1. Participants were asked to visit the laboratory on 3 separate occasions (Days 1, 2, and 5) over 5 days at consistent times of the day. Upon arrival for the first visit (Day 1), participants were requested to complete a series of questions on tobacco use history and awareness of ENDS as well as the Questionnaire on Smoking Urges (QSU) [14]. Participants provided a saliva specimen, a spot urine specimen, and an exhaled breath sample for carbon monoxide (CO) testing. Participants then smoked one of their own cigarettes using the portable CReSS device (Borgwaldt-KC, Richmond, VA). This same device was used by the participant for the remainder of the study. Ten minutes after smoking was completed, participants completed the Cigarette Evaluation Scale (CES) [15], the QSU (post cig use), and provided a second CO sample. Upon departure, participants were asked to take a CReSS device home and smoke any five of their own cigarettes through the device over the next 24 hours.

**Table 1:** Study design

**STUDY DAY**	**1**	**2**	**3**	**4**	**5**
**LAB VISIT**	**1**	**2**			**3**
Lab smoking – tobacco cigarette	X				
Lab smoking – ENDS		X			X
Field smoking – tobacco cigarette	X				
Field smoking – ENDS		X	X	X	
Breath	X	X			X
Saliva	X				X
Urine	X				X
Questionnaires	X	X			X
QSU (pre and post product use)	X	X			X
CES (post product use only)	X	X			X

Participants returned for their 2nd visit and completed the QSU as well as a brief questionnaire about their experience with the CReSS device. They then received a brief description of the ENDS and how to use it, along with a written instruction sheet. A baseline CO reading was obtained and participants were asked to use the ENDS via the CReSS. Neither saliva nor urine samples were collected during the 2nd visit. The remainder of the procedures paralleled Visit 1. Participants were instructed to use the ENDS ONLY (no cigarettes) for the next 72 hours, ending at their 3rd lab visit. They were also instructed to smoke the ENDS through the CReSS device at least 5 times per day over the following 72 hours. Participants were provided a CReSS device, an ENDS, 3 refill cartridges, and instructions to replace the cartridge after about 300 puffs or 24 hours. Participants were also given a product tally sheet to track their product use accurately and a separate form to track their CReSS usage. Participants were instructed to return all study materials including the CReSS device, ENDS unit, and all used and unused cartridges to the laboratory.

Participants arrived 72 hours later for Visit 3 (Day 5), completed the QSU, and were evaluated for compliance with the protocol (no cigarette smoking, defined as CO < 8 ppm per Society for Research on Nicotine and Tobacco (SRNT, 2002). Participants (N = 14) who showed CO ≥8 ppm were reimbursed for their time and excused from the study. Compliant participants completed a brief questionnaire about their experience with the ENDS and the CReSS device. Then, the same biospecimen (saliva and urine) and data collection procedures as Visit 1 were conducted.

This study protocol was reviewed and approved by the Roswell Park Cancer Institute Institutional Review Board. Upon review of a detailed consent form, all participants provided their written informed consent. Participants received a maximum of $50 for completing the study.

### Measures

The CReSSmicro^®^ (Plowshare/Borgwaldt-KC, Richmond VA) was used to record smoking topography and has been shown to provide valid, objective measures of smoking topography [16,17]. A specialized adapter for the ENDS was obtained from Borgwaldt-KC and used in this study. CReSSMicro units were separately calibrated for tobacco cigarettes and ENDS, and dedicated units were used for each product. Topography measures included puff number, puff volume (ml), puff duration (msec), average flow (ml/sec), inter-puff interval (msec), and time and date of smoking. For reporting purposes, duration and inter-puff interval were converted from milliseconds to seconds. We derived total puff volume by summing puff volumes for each cigarette/ENDS.

Saliva specimens were collected using Salivette tubes, and assayed for cotinine using the ELISA method at Salimetrics LLC or at RPCI. Alveolar CO was measured using a Micro 4 Smokerlyzer (Bedfont, Kent, UK). Participants were instructed to hold their breath for 15 seconds before providing a sample of exhaled air. After smoking or ENDS use, 10 minutes elapsed before the next CO reading was taken. CO boost was calculated by subtracting the first reading from the second. Urine specimens were aliquoted and frozen for later analysis.

### Products

The ‘cigarette-like’ “Smoke 51 TRIO” ENDS - 3 piece, First Generation with 11 mg/ml cartridges (Vapor Corp, Miami, FL) was tested in this study, as during the study period it was sold in local shopping mall kiosks. All participants used 11 mg nicotine cartridges with flavor (tobacco, menthol) matched to that of their usual cigarette brand; this concentration was chosen as it was the midpoint of the range offered for this brand at the time. Instructions on ENDS use and proper charging were also provided verbally during the lab session and in writing for participant home reference. The regular cigarettes were the usual brand of the participant and were not provided as part of the study protocol.

### Data analysis

Participant demographics and characteristics were evaluated using basic descriptive analysis. QSU, CES, Topography, and CO differences were all assessed using multivariate repeated measures analysis of variance (ANOVA). Saliva cotinine was analyzed using paired samples t-tests. Log transformations were applied to time to first cigarette and cotinine prior to analysis. Statistical significance was accepted at p < .05, two tailed. Statistical analyses were performed using SPSS version 21 (IBM, Armonk, NY).

## Results

### Participant characteristics

**Figure 1:**
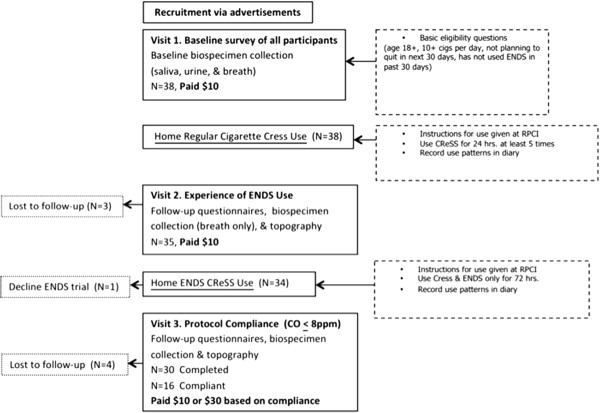
Flowchart of study participation.

Figure 1 is a flowchart of the study from recruitment to final visit. Table 2 shows the demographic layout of the study participants. A total of 38 participants initiated the study with 21.1% (n = 8) being lost to follow-up. Even though 78.9% (n = 30) completed the study, only 42.1% (n = 16) of these 30 completers were actually compliant (CO ≤ 8 ppm) based on study guidelines.

**Table 2:** Demographic characteristics of study participants (N = 38)

**Variable**		**Lost to follow-up after visit 1 or 2 (n = 8)**	**Completed study (n = 30)**		p*
			**Non-compliant (n = 14)**	**Compliant (n = 16)**	
Total percentages		21.1%	36.8%	42.1%	---
Gender	Female	75.0	50.0	62.5	0.53
	Male	25.0	50.0	37.5	
Race	White	50.0	57.1	81.2	0.27
	Black	50.0	42.9	18.8	
% Menthol	Yes	100.0	64.3	75.0	0.21
	No	0.0	35.7	25.0	
Age	Mean	37.9	44.9	45.5	0.36
	(SE)	(5.2)	(2.7)	(3.5)	
Cigarettes per day	Mean	15.4	20.3	16.1	0.25
	(SE)	(2.6)	(2.6)	(1.4)	
Minutes to first cigarette after waking	Geo. Mean	15.9	13.7	13.1	0.94
Baseline cotinine	Geo. Mean	252.6	427.6	338.0	0.15

*Chi-square test for categorical variables; one-way ANOVA for continuous variables.

### ENDS knowledge and beliefs

At baseline, all participants (N = 38) reported they were aware of ENDS. Of these, 29% had ever tried ENDS. The extent of prior experience, outside of use within the past 30-days, was not assessed. Eighty-two percent (N = 31) believed ENDS to be less harmful than regular cigarettes.

### Product usage

At baseline, prior to study commencement, participants reported smoking an average of 17.5 (SD 7.8; Range 10–50) regular cigarettes per day. During the 72-hour field trial, participants were asked to abstain from regular cigarette use and a criterion of CO < = 8 ppm determined compliance. For compliant and non-compliant participants, there was an average 82.0% [V1 - 16.1 cpd; V3 - 2.9 cpd] and average 73.9% [V1 - 20.3 cpd; V3 - 5.3 cpd] reduction in regular cigarette use per day during the ENDS trial period, respectively. Out of those, 2 compliant participants reported no cigarette use at all during the 72-hour period.

### Subjective measures (QSU, CES)

**Figure 2:**
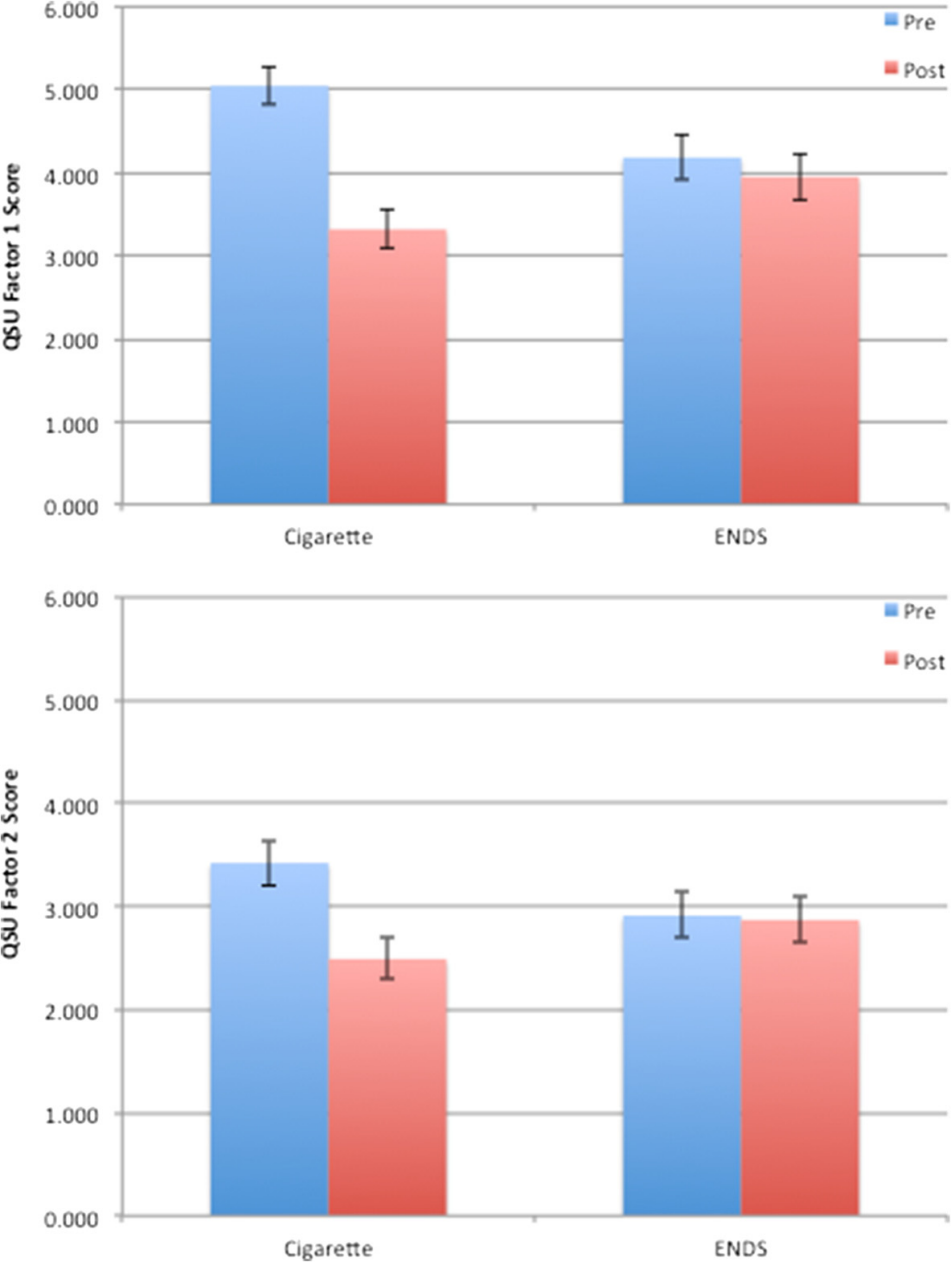
Mean values of questionnaire of smoking urges factor 1 (a) and factor 2 (b) before and after smoking cigarettes and using ENDS.

**Figure 3:**
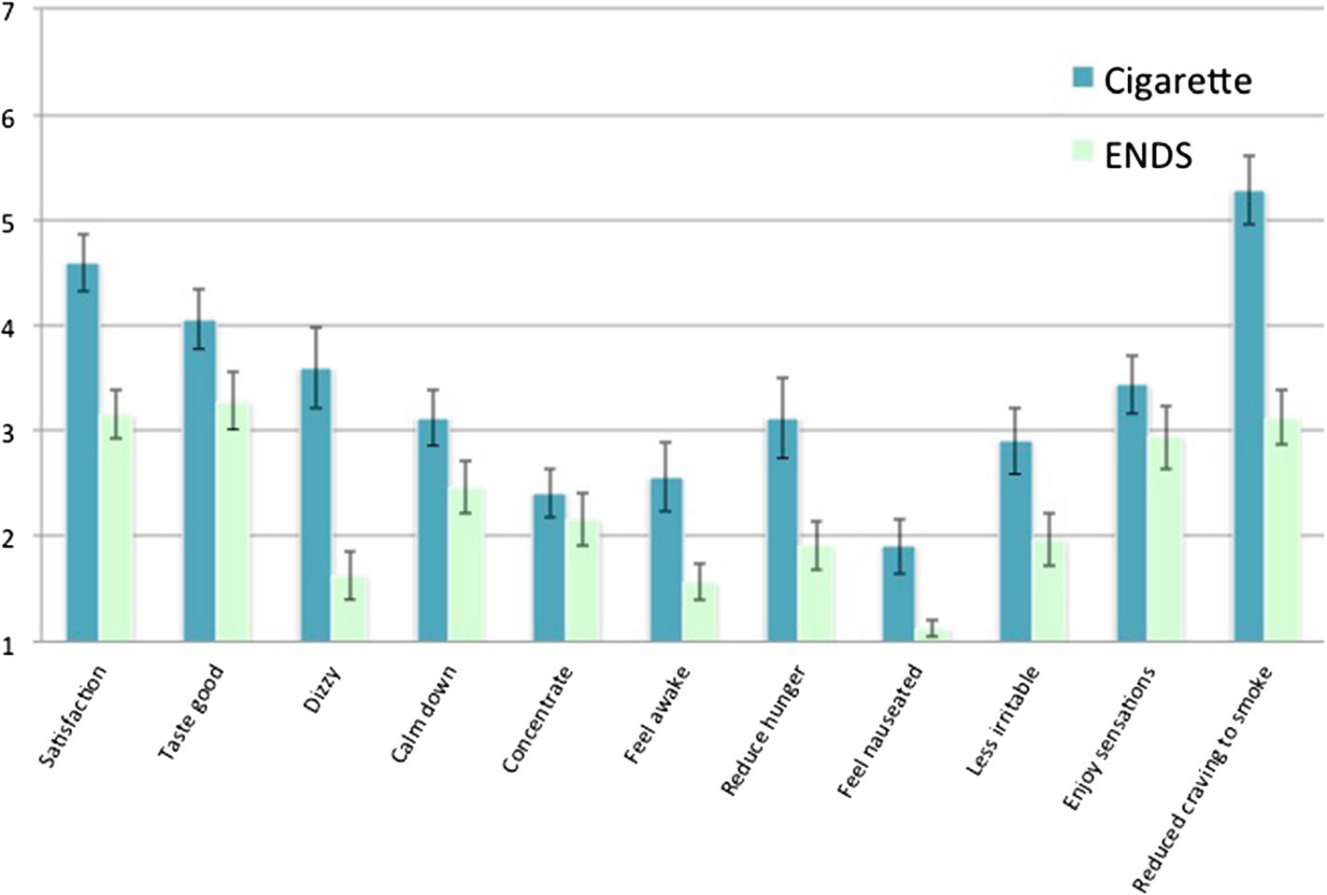
Mean values for cigarette evaluation scale items after smoking cigarettes and using ENDS.

In the laboratory sessions, the observed drop in both QSU Factor 1 & Factor 2 cigarette craving scores (pre-post smoking, n = 32) was statistically significantly greater when smoking own brand cigarettes (Figure 2), compared to ENDS [product * time F(2,30) = 9.62, p = .001]. Indeed, on neither QSU scale did the initial ENDS trial show a significant reduction after use (p’s > .57). Models including gender and age did not show a substantially different pattern of findingsand interaction terms with product and/or time were not statistically significant.Several measures on the Cigarette Evaluation Scale (CES) showed significant differences between cigarettes and ENDS (Figure 3) (n = 32) including satisfaction [F(1,31) = 20.94, p < .001], taste good [F = 4.54, p = .04], dizziness [F = 28.07, p < .001], feeling more awake [F = 12.40, p = .001], reduced hunger for food [F = 22.51, p < .001], increased nausea [F = 10.92, p = .002], feeling less irritable [F = 13.65, p = .001], and reduced craving to smoke cigarettes [F = 28.79, p < .001]. In each case, cigarettes showed significantly higher scores than ENDS. In a separate model, we examined product by age and product by gender interactions. Only two significant effects emerged – women rated cigarettes and ENDS similarly on enjoyment of airway sensation (3.0 vs. 3.4) while men rated cigarettes more highly (4.1 vs. 2.3) [F (1,29) = 8.33, p = .007]. A similar pattern was seen for women (3.8 vs. 3.7) and men (4.4 vs. 2.6) on the ‘taste good’ measure [F = 5.87, p = .02]. The QSU and CES measures were also taken during Visit 3, but due to the low number of completed and compliant participants, are not reported.

### Puffing topography

Equipment failures led to the loss of cigarette topography data on 6 participants and ENDS topography data on 9 participants. A total of 3 participants were missing both. Table 3 shows average puffing topography values by product for both initial laboratory smoking sessions; this includes smoking usual cigarettes and initial experimental ENDS use (n = 18). Overall, when assessed at the laboratory visit, participants showed significantly higher puff counts on cigarettes compared to initial use of ENDS. However, per-puff volume, puff velocity, and peak velocity were significantly higher for experimental ENDS use. We saw no significant differences in puff duration, interval, or total volume drawn. Interactions of product with age and sex were again tested; none emerged as statistically significant.

**Table 3:** Smoking topography mean values for cigarettes and ENDS at the laboratory session

	**Cigarette**		**ENDS**		**ANOVA**	
	**Mean**	**SE**	**Mean**	**SE**	**F (1,17)**	**p**
Puff count (n)	13.2	1.1	8.7	1.6	7.35	0.015
Per-puff volume (ml)	67.5	6.3	118.2	13.3	10.22	0.005
Puff velocity (ml/sec)	36.1	1.8	52.0	4.7	8.00	0.012
Peak velocity (ml/sec)	56.3	4.2	73.4	6.6	4.50	0.049
Duration (sec)	3.0	1.0	3.0	0.8	0.003	0.960
Inter-puff interval (sec)	21.3	6.2	29.6	11.7	0.36	0.555
Total volume (ml)	829.0	51.4	1120.7	320.8	0.84	0.373

### CO and nicotine exposure

Among all participants at baseline, mean exhaled CO was 14.2 ppm (SD = 9.8). Repeated measures ANOVA of exhaled CO (n = 33) showed a significant interaction of product by time [F(1,32) = 84.2, p < .001]. When smoking cigarettes in the laboratory, mean exhaled CO increased from 15.1 ppm (SE 1.7) before smoking to 19.2 ppm (SE 1.9) after smoking. When using ENDS in the laboratory, mean exhaled CO actually declined from 16.4 ppm (SE 2.0) before use to 14.5 ppm (SE 1.9) after use. Analyses controlling for age and gender revealed no statistically significant interactions.

Among all participants at baseline, saliva cotinine had a geometric mean of 344.7 ng/mL; follow up cotinine assessments were made only among those compliant with the protocol (i.e., CO < = 8 ppm at Visit 3). Among compliant participants (N = 16), geometric mean saliva cotinine showed a significant drop between visits 1 (338.0 ng/mL) and 3 (178.4 ng/mL) [t(15) = 4.37, p < .001]. Analyses controlling for age and gender revealed no statistically significant effects.

## Discussion

ENDS are an emerging issue in tobacco control and an evidence base is needed to determine their public health impact. In this small-scale study of brief and initial experience with ENDS, we saw that ENDS use was associated with a decrease in regular cigarettes per day in about half the participants. This effect is not unexpected, as participants had been instructed to stop smoking entirely; however, only 2 participants actually reported smoking no cigarettes during the 72 hour study period. Most, though, reported decreasing their typical cigarette consumption.

Since CO boosts after using ENDS were essentially zero and cotinine levels decreased between Visits 1 and 3 (among compliant participants), the ENDS do not appear to deliver CO (logical as there is no combustion) and appear to deliver less nicotine than cigarettes. The nicotine delivery finding based on cotinine is corroborated by lack of response to subjective indicators of nicotine effects (e.g., dizziness, nausea, cigarette craving reduction). Studies examining nicotine delivery show that first-generation ENDS (such as those studied here) were not very effective at increasing the plasma nicotine concentration levels in naïve research participants relative to cigarettes [18,19]. Under acute testing conditions, while the tested ENDS did not expose users to substantial levels of nicotine or CO, they did suppress nicotine/tobacco abstinence symptom ratings [20]. In a randomized cross-over design study by Farsalinos et al. [21] with 45 experienced ENDS users and 35 smokers, video recorded when using the device, showed that a 20 mg/mL nicotine concentration liquid would be needed in order to deliver nicotine at amounts similar to a tobacco cigarette.

Since most participants were inexperienced using the device for the first time in the laboratory, cigarettes and ENDS also showed distinct smoking patterns; smokers took larger, albeit fewer, puffs of higher velocity on the ENDS. Flow rates (both average and peak) were higher on ENDS compared to cigarettes, paralleling a recent study that showed the airflow rate required to generate aerosol across ENDS was higher than required for tobacco cigarettes [22]. Even though 30% of participants had tried ENDS before, there were no significant topography differences between those who had tried ENDS before and those who had not.

A key issue in assessing the viability of alternatives to cigarettes is consumer perception. A number of studies revealed strong consumer demand for products claiming or implying to reduce health risks [23-27]. Despite this demand, consumers have often rejected such products due to poor taste [28,29]. Sensory characteristics are an important part of cigarette design and replicating these may increase acceptability [30,31]. In the current study, it was clear that smokers did not have equivalent experiences with cigarettes and ENDS. ENDS were rated less positively in taste, satisfaction, and reducing hunger and irritability. Unlike cigarette smoking, we saw no evidence for ENDS reducing cigarette cravings in the laboratory. This was replicated across the two subscales of the QSU and a single item on cigarette craving reduction in the CES. Taken together, this suggests that the product used in this study would likely not serve as a viable full substitute for cigarettes. A caveat remains in that we only tested one brand and there is substantial variability among marketed products in terms of quality and nicotine delivery [2,8]. Moreover, this finding may not generalize across the product class. Indeed, emerging data and product evolution suggest that newer second- and third-generation devices such as e-Go and tank systems deliver nicotine in larger doses and are preferred by experienced vapers [32].

Limitations within this study make replication necessary. Only 38 participants initiated the study, less than half of whom were compliant in abstaining from regular cigarettes for the 72 hour duration. Further, we had intended to complete a more in-depth analysis of topography from the laboratory and home measurements for both cigarettes and ENDS, but equipment failures and noncompliance led to significant data loss that reduced power to detect effects. There were too few compliant participants with viable data to perform analyses of the final visit. Lastly, investigation of the puffing behavior and responses of smokers using an ENDS that matches the nicotine levels generated by the conventional comparator (own brand tobacco cigarettes) is of great interest. Here, the “Smoke 51 TRIO” was an entry level three-piece model with insufficient nicotine delivery. Participants were instructed to abstain from smoking their normal cigarette for 72 hours and only use the ENDS provided. The test of compliance was a CO reading of ≤8 ppm. Because CO levels in smokers can return to non-smoking levels within 24 hours of abstinence, one may hypothesize that participants could attempt to “cheat the system” by smoking their regular cigarettes for the first 48 hours and abstaining for the final 24, or vice versa. However, the clearance rate of CO was not explained to the participants. Additionally, the participants deemed compliant by the CO measurement reported the greatest decrease in cigarette use and increase in ENDS use over the 72 hour period. Thus, in this case, we believe that CO did provide a relatively reliable measure of compliance.

In April 2014, the FDA issued a proposed rule deeming e-cigarettes as tobacco products subject to the Tobacco Control Act [33]. Accumulation of data from studies seeking to understand how consumers use ENDS can only serve to clarify their potential public health risks and benefits.

## Conclusions

Overall, upon initial use, ENDS were smoked more intensively than own brand cigarettes in the laboratory setting. However, ENDS delivered significantly less nicotine, thus participants reported them less satisfying. These findings have implications for the viability of certain first generation ENDS as alternatives to cigarettes. Further research should highlight newer versions of ENDS use over a longer period of time to assess their potential as substitutes for cigarettes.

## Abbreviations

ENDS: Electronic nicotine delivery system; CO: Carbon monoxide; QSU: Questionnaire on smoking urges; CES: Cigarette evaluation scale; SRNT: Society for research on nicotine and tobacco.

## Competing interests

RJO has consulted for the US Food and Drug Administration and World Health Organization on tobacco regulation.

## Authors’ contributions

KJN conducted the participant laboratory visits, participated in data analysis, and drafted the manuscript. KMJ participated in statistical analysis, data interpretation, and drafting the manuscript. RJO conceived and designed the study, performed statistical analysis, and helped draft the manuscript. All authors read and approved the final manuscript.

## References

[B1] Etter JF Bullen C Flouris AD Laugesen M Eissenberg T Electronic nicotine delivery systems: a research agenda Tob Control 2011 20 243 248 10.1136/tc.2010.042168 21415064 PMC3215262

[B2] Trtchounian A Williams M Talbot P Conventional and electronic cigarettes (e-cigarettes) have different smoking characteristics Nicotine Tob Res 2010 12 905 912 10.1093/ntr/ntq114 20644205

[B3] Trtchounian A Talbot P Electronic nicotine delivery systems: is there a need for regulation? Tob Control 2011 20 47 52 10.1136/tc.2010.037259 21139013

[B4] Westenberger BJ Evaluation of e-cigarettes St. Louis: US Food and Drug Administration, Center for Drug Evaluation and Research 2009 http://www.fda.gov/downloads/Drugs/ScienceResearch/UCM173250.pdf

[B5] Uchiyama S Inaba Y Kunugita N Determination of acrolein and other carbonyls in cigarette smoke using coupled silica cartridges impregnated with hydroquinone and 2,4-dinitophenylhydrazine J Chromatogr A 2010 1217 4383 4388 10.1016/j.chroma.2010.04.056 20483418

[B6] Goniewicz ML Knysak J Gawron M Kosmider L Sobczak A Kurek J Prokopowicz A Jablonska-Czapla M Rosik-Dulewska C Havel C Jacob P Goniewicz ML Knysak J Gawron M Kosmider L Sobczak A Kurek J Prokopowicz A Jablonska-Czapla M Rosik-Dulewska C Havel C Jacob P III Benowitz N Levels of selected carcinogens and toxicants in vapour from electronic cigarettes Tob Control 2013 Epub ahead of print 10.1136/tobaccocontrol-2012-050859 PMC4154473 23467656

[B7] Hua M Yip H Talbot P Mining data on usage of electronic nicotine delivery systems (ENDS) from YouTube videos Tob Control 2013 22 103 106 10.1136/tobaccocontrol-2011-050226 22116832

[B8] Goniewicz ML Kuma T Gawron M Knysak J Kosmider L Nicotine levels in electronic cigarettes Nicotine Tob Res 2013 15 158 166 10.1093/ntr/nts103 22529223

[B9] Adkison SE O’Connor RJ Bansal-Travers M Hyland A Borland R Yong HH Cummings KM McNeill A Thrasher JF Hammond D Fong GT Electronic nicotine delivery systems: international tobacco control four-country survey Am J Prev Med 2013 44 207 215 10.1016/j.amepre.2012.10.018 23415116 PMC3627474

[B10] Polosa R Caponnetto P Morjaria JB Papale G Campagna D Russo C Effect of an electronic nicotine delivery device (e-Cigarette) on smoking reduction and cessation: a prospective 6-month pilot study BMC Public Health 2011 11 786 10.1186/1471-2458-11-786 21989407 PMC3203079

[B11] Goniewicz ML Lingas EO Hajek P Patterns of electronic cigarette use and user beliefs about their safety and benefits: An Internet survey Drug Alcohol Rev 2012 Epub ahead of print 10.1111/j.1465-3362.2012.00512.x PMC3530631 22994631

[B12] Caponnetto P Campagna D Cibella F Morjaria JB Caruso M Russo C Polosa R EffiCiency and Safety of an eLectronic cigarette (ECLAT) as tobacco cigarettes substitute: a prospective 12-month randomized control design study PLoS ONE 2013 8 e66317 10.1371/journal.pone.0066317 23826093 PMC3691171

[B13] Bullen C McRobbie H Thornley S Glover M Lin R Laugesen M Effect of an electronic nicotine delivery device (e cigarette) on desire to smoke and withdrawal, user preferences and nicotine delivery: randomized cross-over trial Tob Control 2010 19 98 103 10.1136/tc.2009.031567 20378585

[B14] Tiffany ST Drobes DJ The development and initial validation of a questionnaire on smoking urges Br J Addict 1991 86 1467 1476 10.1111/j.1360-0443.1991.tb01732.x 1777741

[B15] Westman E Levin E Rose J Smoking while wearing the nicotine patch: Is smoking satisfying or harmful? Clin Res 1992 40 871A

[B16] Hammond D Fong GT Cummings KM Hyland A Smoking topography, brand switching, and nicotine delivery: results from an in vivo study Cancer Epidemiol Biomarkers Prev 2005 14 1370 1375 10.1158/1055-9965.EPI-04-0498 15941943

[B17] Blank MD Disharoon S Eissenberg T Comparison of methods for measurement of smoking behavior: mouthpiece-based computerized devices versus direct observation Nicotine Tob Res 2009 11 896 903 10.1093/ntr/ntp083 19525207 PMC2699933

[B18] Vansickel AR Eissenberg T Electronic cigarettes: effective nicotine delivery after acute administration Nicotine Tob Res 2013 15 267 270 10.1093/ntr/ntr316 22311962 PMC3524053

[B19] Bullen C Whittaker R Walker N Wallace-Bell M Pre-quitting nicotine replacement therapy: findings from a pilot study Tob Induc Dis 2006 3 35 40 10.1186/1617-9625-3-2-35 19570295 PMC2633370

[B20] Vansickel AR Cobb CO Weaver MF Eissenberg TE A clinical laboratory model for evaluating the acute effects of electronic “cigarettes”: nicotine delivery profile and cardiovascular and subjective effects Cancer Epidemiol Biomarkers Prev 2010 19 2945 2953 10.1158/1055-9965.EPI-10-0288 PMC2919621 20647410

[B21] Farsalinos KE Romagna G Tsiapras D Kyrzopoulos S Voudris V Evaluation of electronic cigarette use (vaping) topography and estimation of liquid consumption: implications for research protocol standards definition and for public health authorities’ regulation Int J Environ Res Public Health 2013 10 2500 2514 10.3390/ijerph10062500 23778060 PMC3717749

[B22] Williams M Talbot P Variability among electronic cigarettes in the pressure drop, airflow rate, and aerosol production Nicotine Tob Res 2011 13 1276 1283 10.1093/ntr/ntr164 21994335

[B23] Cummings KM Hyland A Giovino GA Hastrup JL Bauer JE Bansal MA Are smokers adequately informed about the health risks of smoking and medicinal nicotine? Nicotine Tob Res 2004 6 Suppl 3 333 340 10.1080/14622200412331320734 15799596

[B24] Hamilton WL Norton G Ouellette TK Rhodes WM Kling R Connolly GN Smokers’ responses to advertisements for regular and light cigarettes and potential reduced exposure tobacco products Nicotine Tob Res 2004 6 3 353 362 10.1080/14622200412331320752 15799598

[B25] Parascandola M Hurd AL Augustson E Consumer awareness and attitudes related to new potential reduced-exposure tobacco products Am J Health Behav 2008 32 431 437 18092903 10.5555/ajhb.2008.32.4.431

[B26] Shiffman S Pillitteri JL Burton SL Di Marino ME Smoker and ex-smoker reactions to cigarettes claiming reduced risk Tob Control 2004 13 78 84 10.1136/tc.2003.005272 14985602 PMC1747811

[B27] Shiffman S Hughes JR Ferguson SG Pillitteri JL Gitchell JG Burton SL Smokers’ interest in using nicotine replacement to aid smoking reduction Nicotine Tob Res 2007 9 1177 1182 10.1080/14622200701648441 17978992

[B28] Fairchild A Colgrove J Out of the ashes: the life, death, and rebirth of the “safer” cigarette in the United States Am J Public Health 2004 94 192 204 10.2105/AJPH.94.2.192 14759927 PMC1448228

[B29] Ferris Wayne G Connolly GN Application, function, and effects of menthol in cigarettes: a survey of tobacco industry documents Nicotine Tob Res 2004 6 Suppl 1 43 54 10.1080/14622203310001649513 14982708

[B30] Carpenter CM Wayne GF Connolly GN The role of sensory perception in the development and targeting of tobacco products Addiction 2007 102 136 147 10.1111/j.1360-0443.2006.01649.x 17207131

[B31] Wayne GF Connolly GN How cigarette design can affect youth initiation into smoking: Camel cigarettes 1983–93 Tob Control 2002 11 Suppl 1 I32 I39 11893812 10.1136/tc.11.suppl_1.i32 PMC1766065

[B32] Dawkins L Turner J Roberts A Soar K ‘Vaping’ profiles and preferences: an online survey of electronic cigarette users Addiction 2013 108 1115 1125 10.1111/add.12150 23551515

[B33] Food and Drug Administration Deeming Tobacco Products to be subject to the Federal Food, Drug, and Cosmetic Act, as amended by the Family Smoking Prevention and Tobacco Control Act; Regulations on the Sale and Distribution of Tobacco Products and Required Warning Statements for Tobacco Products Fed Regist 2014 79 80 23142 23207 27192730

